# Commentary: Effects of Video Game Training on Measures of Selective Attention and Working Memory in Older Adults: Results from a Randomized Controlled Trial

**DOI:** 10.3389/fnagi.2017.00442

**Published:** 2018-01-10

**Authors:** Elzbieta Szelag

**Affiliations:** Laboratory of Neuropsychology, Department of Neurophysiology, Nencki Institute of Experimental Biology, Polish Academy of Sciences, Warsaw, Poland

**Keywords:** healthy aging, neurorehabilitation, cognitive training, video games

The article by Ballesteros and colleagues provides a fresh approach to the cognitive neuroscience of aging with a central focus on enhancement of cognitive functions by a video game training in normal healthy elderly.

In the Twenty-first century the interest of researchers on beneficial effects of various cognitive therapies in amelioration of the mental power in elderly has grown rapidly with the increasing population of older people and it is the topic of many scientific papers, meta-analyses and review articles. As aging triggers progressive loss in multiple cognitive systems (Szelag et al., 2010; Kennedy and Raz, 2015), increasing proportion of elderly has suggested that more individuals may probably suffer from deficient mental activity. By emphasizing this demographic perspective, there has been a great explosion of studies devoted possible ways to preserve or enhance the mental power in elderly. As this line of research is of a great practical relevance, neurorehabilitation in elderly constitutes a challenging topic in contemporary neuroscience.

On this background Ballesteros et al. provide an important, well-structured and interesting state-of-the-art summary on the existing cognitive training studies focused on non-action computer video games. Their treatment model bases on a comparison of cognitive benefits following the application of non-action games from the commercial platform Lumosity (experimental group) and simulation strategy games (active controls). Transfer of video-game gains is studied with two selective attention (cross-modal oddball and Stroop-negative priming) and two working memory tasks (Corsi blocks and N-back), considering self-report data on motivation, engagement and expectations in two compared groups. The main finding is that the Lumosity did not result in greater cognitive improvements than the intervention based on simulation games. Thus, video-game training provides modest benefits for untrained cognitive tasks.

I would like to express some methodological comments on this experimentation raised also in my review of this study. These comments may be important for future neurorehabilitation studies.

First, in training studies the inclusion of the non-active (passive) control group seems necessary, however, it is missing in the study by Ballesteros et al. It creates some methodological imbalance and may lead to unclear conclusions. Such extra passive controls should be tested twice in a time distance corresponding to the intervention duration. It provides evidence on possible changes in cognitive performance caused by the measurement repeated twice in a relatively short time (before *vs*. after the intervention, in this study in ca. 12 weeks). In my opinion such effect cannot be avoided, even using the alternative test versions because of memorized task items or a strategy learning. The other influences may be caused by subjects' participation in the training protocol. Therefore, such extra control group allows to control for these nonspecific influences. Without the convincing control conditions any conclusion on training-related improvement may be misleading, because a contribution of nonspecific factors may be reflected even in the marginal improvements, as reported here.

Second, the follow-up assessment which is also missing in this paper seems necessary as it allows to verify the stability of achieved cognitive gains (even modest) or a possibility of distant gains resulting from accumulation of neuroplastic changes in the brain. Without the follow-up assessment one cannot conclude whether the obtained intervention-related benefits (even marginal) are transient or more stable, thus, based probably on reorganization within the neural network.

The most important issue concerns the discussion and interpretation of data which focus on the behavioral level only and neglect any explanation on underlying mechanisms. A challenging problem in cognitive training studies is to define the neural mechanisms (or processes) that could account for training-related changes. Despite the statement that in elderly the application of non-action video games may influence modestly selective attention and working memory, one should agree that it influences the underlying neural network because the training-related gains are rooted in neuroplastic reorganization within the brain. Such approach is often ignored among researchers, leading to difficulties in understanding why the training-related changes may be expected.

An important question arising from this study is: *What neural mechanisms (or processes) may underlie the training-related benefits? One* candidate indicated in Ballesteros's paper are executive functions that are high demand meta-cognitive processes that guide the optimization of goal-oriented behaviors (Etnier and Chang, 2009). The question is whether we could define also more general functions that account for these executive functions. In this commentary I aim at addressing this conceptual gap.

A number of literature studies have indicated the patterning in time as one of characteristic features of human cognition because many mental functions are rooted in the exact temporal template (Pöppel, 1997, 2009; Szelag et al., 2015). The idea of time being incorporated in cognition is not new, indeed, recently the large increase of research in that field is observed (Block and Grondin, 2014). Temporal processing may be measured on several levels, some tens of millisecond range is evidenced to underlie temporal resolution that controls sequential processing (Wittmann, 1999). An overlapping of declined timing and deteriorated cognition has been reported in many clinical groups, including normal elderly population (Salthouse, 1996, 2009; Szelag et al., 2011; Teixeira et al., 2013).

In our previous report we extended the relation “*timing-cognition*,” indicating correlations between the efficiency of temporal resolution and executive functions (Nowak et al., 2016). It suggests a common neural mechanisms underlying these two mental functions. Thus, the deteriorated neural clock leads to declined executive functions in late adulthood. From the evidence briefly reported here we postulate that executive functions are governed by a central timing processor. Furthermore, we indicated that training in timing ameliorates mental functioning in elderly (Szelag and Skolimowska, 2012).

On a basis of these evidence one could reinterpret the merits of this paper. Age-related disruption of timing leads to declined cognition. Playing of video-games influences cognitive and executive functions because it might enhance the temporal resolution of the system. As a researcher in the field of timing, I am on the opinion that studies focused on cognitive skills in isolation, without any analysis of neural substrates cannot give a complete picture of the mental power in elderly brains.

## Author contributions

The author confirms being the sole contributor of this work and approved it for publication.

### Conflict of interest statement

The author declares that the research was conducted in the absence of any commercial or financial relationships that could be construed as a potential conflict of interest.
